# A −436C>A Polymorphism in the Human *FAS* Gene Promoter Associated with Severe Childhood Malaria

**DOI:** 10.1371/journal.pgen.1002066

**Published:** 2011-05-19

**Authors:** Kathrin Schuldt, Cosima C. Kretz, Christian Timmann, Jürgen Sievertsen, Christa Ehmen, Claudia Esser, Wibke Loag, Daniel Ansong, Carmen Dering, Jennifer Evans, Andreas Ziegler, Jürgen May, Peter H. Krammer, Tsiri Agbenyega, Rolf D. Horstmann

**Affiliations:** 1 Department of Molecular Medicine, Bernhard Nocht Institute for Tropical Medicine, Hamburg, Germany; 2 Institute of Medical Biometry and Statistics, University at Lübeck, University Hospital Schleswig-Holstein, Lübeck, Germany; 3 Division of Immunogenetics, German Cancer Research Centre, Heidelberg, Germany; 4 Infectious Disease Epidemiology Group, Bernhard Nocht Institute for Tropical Medicine, Hamburg, Germany; 5 School of Medical Sciences, Kwame Nkrumah University of Science and Technology, Kumasi, Ghana; University College London, United Kingdom

## Abstract

Human genetics and immune responses are considered to critically influence the outcome of malaria infections including life-threatening syndromes caused by *Plasmodium falciparum*. An important role in immune regulation is assigned to the apoptosis-signaling cell surface receptor CD95 (Fas, APO-1), encoded by the gene *FAS*. Here, a candidate-gene association study including variant discovery at the *FAS* gene locus was carried out in a case-control group comprising 1,195 pediatric cases of severe falciparum malaria and 769 unaffected controls from a region highly endemic for malaria in Ghana, West Africa. We found the A allele of c.−436C>A (rs9658676) located in the promoter region of *FAS* to be significantly associated with protection from severe childhood malaria (odds ratio 0.71, 95% confidence interval 0.58–0.88, p_empirical_ = 0.02) and confirmed this finding in a replication group of 1,412 additional severe malaria cases and 2,659 community controls from the same geographic area. The combined analysis resulted in an odds ratio of 0.71 (95% confidence interval 0.62–0.80, p = 1.8×10^−7^, n = 6035). The association applied to c.−436AA homozygotes (odds ratio 0.47, 95% confidence interval 0.36–0.60) and to a lesser extent to c.−436AC heterozygotes (odds ratio 0.73, 95% confidence interval 0.63–0.84), and also to all phenotypic subgroups studied, including severe malaria anemia, cerebral malaria, and other malaria complications. Quantitative FACS analyses assessing CD95 surface expression of peripheral blood mononuclear cells of naïve donors showed a significantly higher proportion of CD69^+^CD95^+^ cells among persons homozygous for the protective A allele compared to AC heterozygotes and CC homozygotes, indicating a functional role of the associated CD95 variant, possibly in supporting lymphocyte apoptosis.

## Author Summary

Severe malaria caused by infection with the protozoan parasite *Plasmodium falciparum* is a major health burden, causing approximately one million fatalities annually, predominantly among young children in Sub-Saharan Africa. The occurrence of severe malaria may depend on a complex interplay of transmission dynamics and the development of a protective immune response but also on heritable differences in the susceptibility to the disease. In two large studies including a total of 2,607 affected children and 3,428 apparently healthy individuals from Ghana, West Africa, we investigated genetic variants of the *FAS* gene, which encodes CD95, a molecule critically involved in the programmed cell death of lymphocytes. We found that a single nucleotide variant in the *FAS* promoter was associated with a 29%–reduced risk of developing severe malaria. In individuals carrying two copies of the protective allele, a higher proportion of activated lymphocytes was found to express CD95. These findings indicate that a predisposition to an increased expression of CD95 may help to protect from severe malaria, possibly by rendering activated T-lymphocytes more susceptible to programmed cell death.

## Introduction

Severe malaria caused by infection with the protozoan parasite *Plasmodium falciparum* worldwide causes approximately one million fatalities annually, mostly among children in Sub-Saharan Africa [1]. The clinical picture of severe malaria is characterized by a range of distinct but overlapping syndromes including severe anemia, coma and convulsions, respiratory distress, and others [2]. The variability of the phenotype may be explained by differences in transmission dynamics and the development of the host's immune reactions but also by heritable differences in susceptibility to the disease [3].

The gene *FAS* (*TNFRSF6*, *APT1*) at chromosome 10q24.1 encodes the cell surface receptor CD95 (Fas, APO-1), known as the prototypic death receptor [4], [5]. CD95 is a widely expressed molecule with the ability to transduce signals that promote cell death by apoptosis [6]. The CD95-mediated proapoptotic function is triggered by its natural ligand, CD95L, which predominantly is expressed on cells of the T-cell lineage but also acts in a functional soluble form [7], [8]. The CD95/CD95L system plays a key role in T-cell apoptosis and immune homeostasis as indicated by the induction of lymphoproliferation and autoimmunity in patients with mutations in either the receptor or its ligand [9]. In the case of infections with persistent antigenic challenge, programmed cell death via CD95/CD95L signaling is involved in the elimination of activated lymphocytes, a process indispensable to prevent vital tissues from collateral damage caused by prolonged immune activation [10], [11].

Indications for an implication of CD95 during severe malaria episodes have been gained from several studies. For instance, lymphocytes from malaria patients were found to express markers of apoptosis and are susceptible to activation-induced cell death (AICD) *in vitro*, and serum samples of patients with *P. falciparum* malaria show elevated levels of soluble CD95L as compared to healthy subjects [12], [13]. More specifically, as demonstrated by Balde and colleagues, the exposure of peripheral blood mononuclear cells (PBMC) to *P. falciparum* extract caused a marked increase in the expression of functional CD95 [14]. To date, two studies addressing genome-wide transcriptional changes in blood cells from patients with symptomatic malaria have been conducted [15], [16]. In Cameroon, the expression profile in PBMC fractions was assessed using samples from adults diagnosed for severe malaria, whereas in a study from Kenya host gene expression was determined in cells from whole blood derived from acute pediatric cases. Although major differences exist between the two experimental designs, both studies detected a significant increase in the expression of CD95 in circulating blood cells during an acute falciparum malaria episode. These findings suggest a role for CD95 in the immune response to infections with *P. falciparum*, in which its precise function has yet to be defined.

In the present study, we sought to elucidate the impact of *FAS* genetic variants on malaria susceptibility by conducting a candidate-gene association study, which involved a variant screen through re-sequencing and genotyping of selected variants in the *FAS* gene in a sample set including 1195 severe malaria cases and 769 apparently healthy controls recruited in Ghana, West Africa. In addition, the impact of variant c.−436C>A was further investigated in a replication study from the same geographical area, including 1412 children with severe malaria and 2659 community controls. With regard to the association of variant c.−436C>A (rs9658676) with protection from severe malaria in our study we characterized the allele-dependent CD95 surface expression of PBMCs by quantitative fluorescence-activated cell sorting (FACS).

## Results

### Variants studied

At total of 19 variants were analyzed in the initial case-control study. Genotypes for 14 of these polymorphisms were derived using the Affymetrix Genome-Wide Human SNP Array 6.0. Additional five variants were selected for genotyping after they were identified by re-sequencing the *FAS* gene including exonic and regulatory regions in 46 individuals from our study group. Among the 16 single nucleotide polymorphisms (SNPs) detected by re-sequencing, two polymorphisms, c.−671G>A (rs1800682) in the 5′-flanking region and c.46G>A (rs3218619) in exon 1 had previously been shown to exhibit a substantial effect on the expression and function of the receptor [17], [18]. Hence, both were selected for genotyping aside from c.365C>T (rs3218614) because it results in a non-synonymous amino acid exchange (T122I) in the receptor as well as c.−436C>A (rs9658676) and c.141G>A (rs3218621) because they showed differences in estimated MAFs between cases and controls of 0.16 and 0.15, respectively. One SNP, c.*978C>T, located in the 3′-UTR of the gene, was newly identified. Due to an estimated MAF of 0.02 this SNP was not selected for genotyping. The SNPs found, their localizations, and estimated allele frequencies are summarized in Table S1.

### Association of *FAS* variant c.−436C>A with severe malaria

Genotype frequencies did not deviate from Hardy-Weinberg Equilibrium (HWE) (p>0.01) except for c.334+46C>T, which in the case group showed a deviation with p = 6.2×10^−4^. When analysing genotypes in the first case-control sample (n = 1964) a significant result was obtained for the promoter variant c.−436C>A in the trend test (p = 1.3×10^−3^). After adjustment for multiple testing, the association of c.−436C>A in the logistic regression analysis remained significant for the additive model of inheritance (p_empirical_ = 0.02) and reached borderline significance for the dominant model (p_empirical_ = 0.05) (Table 1). The A allele of c.−436C>A was found to be more frequent among controls than among cases and thus was associated with protection. The odds ratios (ORs) of c.−436C>A were homogeneous in the three ethnic groups included in the study group (p = 0.92).

**Table 1:** Logistic regression results for SNPs of the *FAS* gene locus^a^.

SNP ID	Position^b^	MinorAllele	Cases MAF	Controls MAF	p-value^c^ trend test	Additive Model		Dominant Model		Recessive Model	
						OR^d^ (95% CI^e^)	Empirical p-value^f^	OR^d^ (95% CI^e^)	Empirical p-value^f^	OR^d^ (95% CI^e^)	Empirical p-value^f^
rs7916294	c.−6308	A	0.48	0.47	0.47	1.05	0.99	1.10	0.99	1.03	1
						(0.92–1.96)		(0.90–1.35)		(0.83–1.28)	
rs1800682	c.−671	A	0.21	0.22	0.64	0.95	0.99	0.95	1.0	0.92	1
						(0.81–1.12)		(0.77–1.16)		(0.59–1.44)	
rs9658676	c.−436	A	0.09	0.12	1.3×10^−3^	0.71	0.02	0.71	0.05	0.43	0.47
						(0.58–0.88)		(0.56–0.90)		(0.19–1.00)	
rs10509561	c.30+1249	A	0.21	0.22	0.41	0.94	0.94	0.96	1	0.78	0.98
						(0.80–1.09)		(0.80–1.16)		(0.51–1.19)	
rs7097467	c.30+2581	C	0.17	0.16	0.28	1.11	0.96	1.19	0.68	0.71	0.96
						(0.93–1.34)		(0.97–1.46)		(0.39–1.30)	
rs7097572	c.30+2597	C	0.17	0.17	0.80	0.98	1	1.01	1	0.71	0.96
						(0.82–1.16)		(0.83–1.23)		(0.42–1.22)	
rs1926196	c.30+3085	C	0.17	0.17	0.80	0.98	1	1.02	1	0.69	0.91
						(0.82–1.16)		(0.84–1.24)		(0.40–1.18)	
rs9658702	c.31-5541	A	0.14	0.15	0.63	0.95	1	0.96	1	0.82	1
						(0.79–1.15)		(0.78–1.18)		(0.44–1.53)	
rs3218619	c.46	A	0.14	0.15	0.84	1.03	1	1.04	1	0.94	1
						(0.86–1.23)		(0.85–1.28)		(0.47–1.84)	
rs3218621	c.141	A	0.11	0.11	0.29	0.90	0.97	0.90	0.98	0.80	1
						(0.74–1.10)		(0.73–1.12)		(0.38–1.67)	
rs9658733	c.196+338	T	0.15	0.13	0.12	1.15	0.77	1.14	0.93	1.63	0.91
						(0.96–1.39)		(0.93–1.40)		(0.81–3.3)	
rs2296601	c.334+46	T	0.19	0.18	0.39	1.08	1	0.98	1	2.2	0.04
						(0.91–1.28)		(0.80–1.20)		(1.29–3.8)	
rs3218614	c.365	T	0.05	0.06	0.56	0.91	0.92	0.87	0.99	NA	NA
						(0.81.28)		(0.65–1.17)			
rs2234978	c.642	C	0.32	0.34	0.21	0.92	0.94	0.92	0.99	0.84	0.98
						(0.80–1.05)		(0.77–1.10)		(0.63–1.12)	
rs1468063	c.*1084	T	0.22	0.20	0.24	1.10	0.94	1.05	1	1.63	0.36
						(0.94–1.29)		(0.87–1.27)		(1.03–2.58)	
rs2862833	c.*1422	G	0.46	0.45	0.84	1.01	1	1.05	1	0.96	1
						(0.89–1.15)		(0.86–1.28)		(0.77–1.21)	
rs1800623	c.*1876	G	0.33	0.35	0.15	0.91	0.85	0.92	0.99	0.81	0.82
						(0.79–1.04)		(0.77–1.11)		(0.61–1.07)	
rs4934435	c.*2189	G	0.33	0.35	0.15	0.91	0.84	0.92	0.99	0.81	0.84
						(0.79–1.04)		(0.76–1.10)		(0.61–1.07)	
rs7915235	c.*4890	A	0.21	0.19	0.15	1.13	0.8	1.09	0.99	1.63	0.42
						(0.96–1.32)		(0.90–1.32)		(1.01–2.62)	

a Results of SNPs are based on genotypes from 1195 cases and 769 controls.

b Position in Transcript NM_000043.3;

c uncorrected;

d OR, Odds ratio;

e CI, Confidence interval;

f empirical p-value based on 10,000 permutations of case-control status using the maxT procedure.

**Figure 1:**
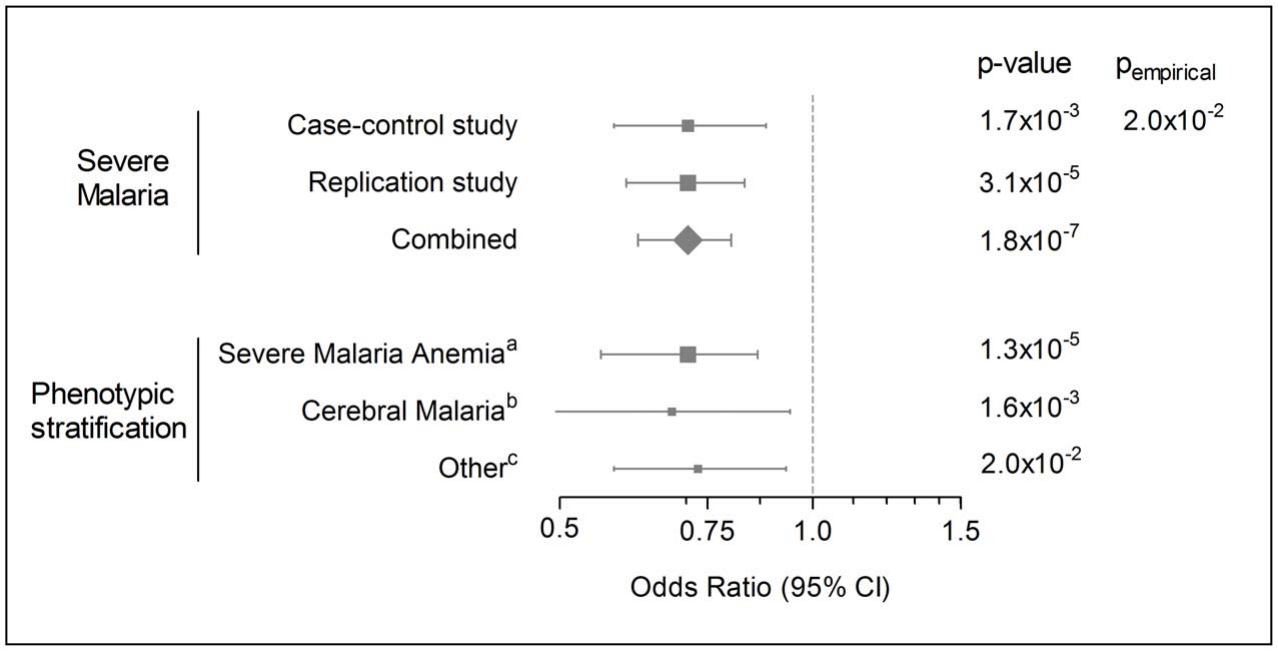
Forest plot of *FAS* c.−436.C>A association with severe malaria and sub-phenotypes. Odds ratios were calculated assuming the additive model of inheritance; symbol sizes represent relative numbers of individuals. ^a^Combined result for severe malaria anemia with or without additional complications including cerebral malaria (n = 1626). ^b^Combined result for cerebral malaria with or without additional complications including severe anemia (n = 569). ^c^Severe malaria complications not including severe anemia or cerebral malaria (hyperlactatemia, 100%; prostration, 83%; hyperparasitemia, 56%; acidosis, 54%; respiratory distress, 27%, n = 518).

The association of c.−436C>A was confirmed in a replication group of 1412 additional severe-malaria children and 2659 controls (Figure 1). Like in the initial study group, strongest evidence for association was obtained when applying the additive inheritance model (OR 0.71, 95% confidence interval (CI) 0.60–0.83, p = 3.1×10^−5^). Combining the results of the two case-control groups yielded a significance level of p = 1.8×10^−7^ (fixed effect model; OR 0.71, 95% CI 0.62–0.80, Figure 1), with no evidence for heterogeneity of the effects (p = 0.98, Cochran's Q statistic).

With a combined OR of 0.47 (95% CI 0.36–0.60) c.−436AA homozygous individuals appear to be protected to a greater extent than heterozygous individuals (OR 0.73, 95% CI 0.63–0.84). The data are compatible with an additive inheritance model (approach of Bagos, p = 0.498), whereas both the dominant and recessive genetic models were rejected (p = 0.044 and p = 0.0003, respectively).

With the aim of specifying the association of marker c.−436C>A with regard to severe malaria pathology, a stratified analysis for clinically distinct subgroups was carried out. After combining the results from both study groups ORs for the additive mode of inheritance were found to be similar for the clinical subgroups of cerebral malaria, severe anemia, and collectively other complications independent of sample size differences (Figure 1). Combined ORs ranged from 0.68 for cerebral malaria cases (95% CI 0.42–0.94, p = 1.6×10^−3^; n_cases_ = 569) to a maximum of 0.73 when including cases with complications such as hyperlactatemia, prostration, and hyperparasitemia collectively (95% CI 0.58–0.92, p = 0.02; n_cases_ = 518). The results pointed towards a protective effect of the A allele in all sub-phenotypes of severe malaria studied. When analyzing log-transformed parasite densities in the combined case groups (n = 2353), no significant differences between the three genotypic groups were detected (p = 0.67).

In order to evaluate a possible association of c.−436C>A with mild malaria, a quantitative transmission disequilibrium test (qTDT) was performed with the numbers of uncomplicated malaria episodes experienced by 390 Ghanaian siblings over a period of 31 weeks [19]. No significant distortion of transmission was found (p = 0.96). Likewise, no indication for an association with peripheral-blood parasite densities was detected when using the 75th percentile of the siblings' parasite counts (p = 0.55). Given the clinical and parasitological data, the statistical power to reveal an influence of c.−436C>A on these two phenotypes was 45% and 85%, respectively.

### Associations with severe malaria of the other *FAS* variants studied

Of the additional 18 variants studies (Table 1), only c.334+46C>T showed a disease association in the initial case-control group, p_empirical_ being 0.04. This was seen only under a recessive model of inheritance. As the significant findings obtained with c.−436C>A were limited to the assumption of an additive inheritance mode, the association of c.334+46C>T being of borderline significance was not pursued further.

### Linkage disequilibrium and haplotype analysis

**Figure 2:**
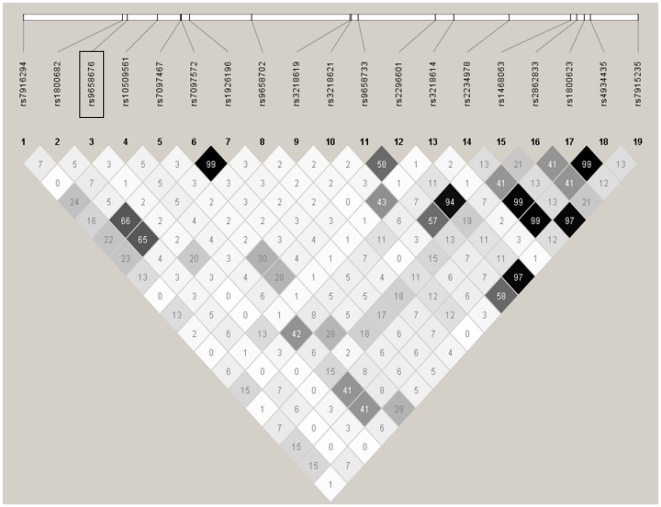
LD structure at the *FAS* gene locus. LD pattern is represented by pairwise r^2^ values between SNPs with MAF >5% based on genotypes from 1195 cases and 769 controls. r^2^ values (×100) for each comparison are given in the squares. White squares represent r^2^ values equal to 0. Squares that are shades of grey represent r^2^ values between 0 and 1.

The analysis of the genomic structure at the *FAS* locus displayed remarkably low pairwise linkage disequilibria between markers, particularly in the promoter region. The variant c.−436C>A shares a maximum r^2^-value of 0.20 with c.46G>A, located 12.6 kb apart in exon 2 of *FAS* (Figure 2). Towards the 3′-end of the gene correlation between variants increases, especially for non-coding variants. Within the entire region of approximately 35 kb frequencies of inferred haplotypes were compared in a score test adjusted for age, gender, and ethnicity. Score statistics including full haplotypes with frequencies of >5% did not reveal any significant association with disease (global p-values: additive model p = 0.15, dominant p = 0.32, recessive p = 0.45; haplotype-specific results in Table S2). However, evidence for a haplotypic association was observed when analyzing sub-haplotypes under the additive and dominant models, where a haplotype comprising the three alleles c.−436A, c.30+1249A, and c.30+2581T was found to be associated with protection from severe malaria (additive global p = 0.018, dominant global p = 2.2×10^−4^; Figure S1).

### CD95 surface expression of peripheral blood mononuclear cells

**Figure 3:**
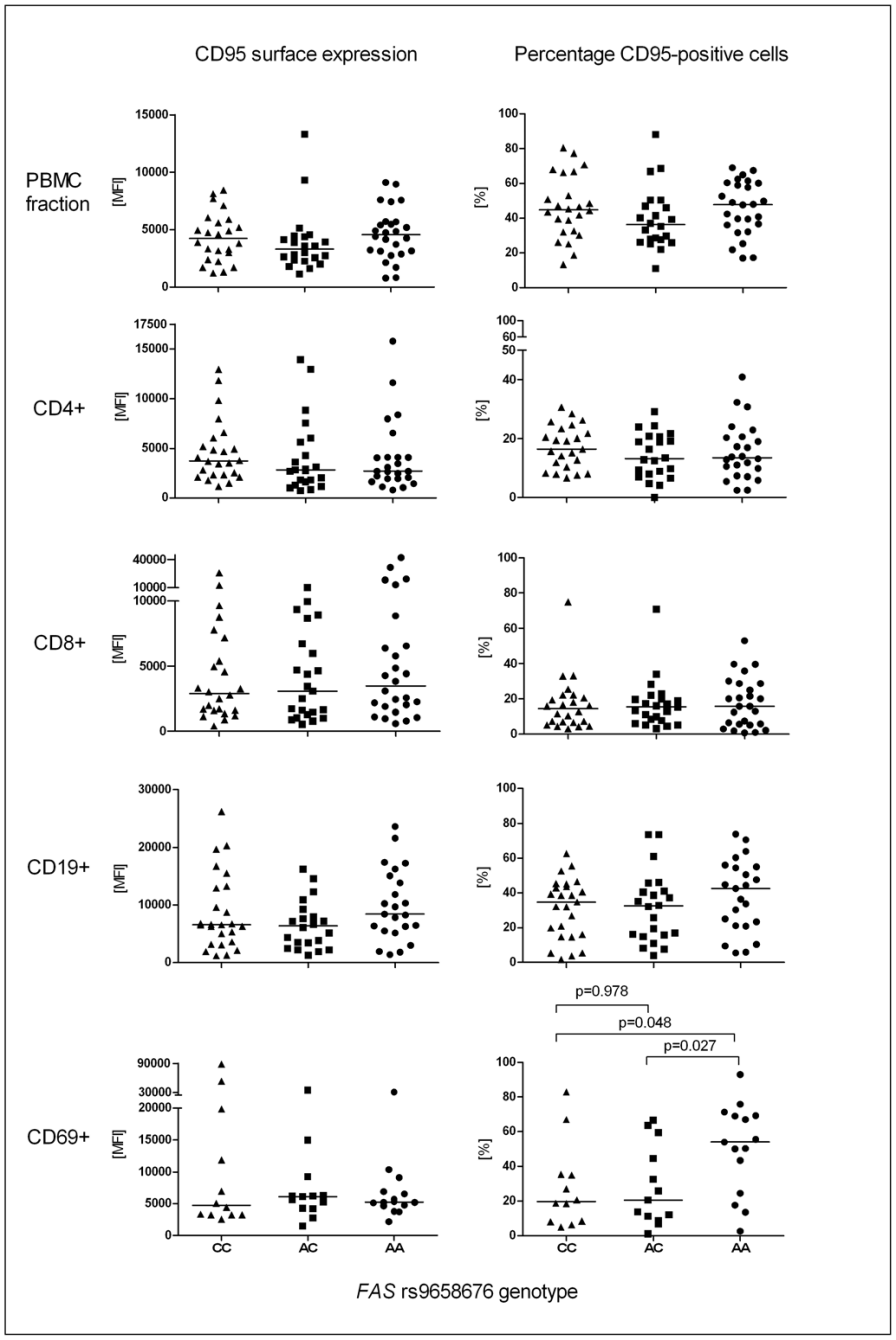
*FAS* c.−436C>A dependent surface expression of CD95 on peripheral blood mononuclear cells. CD95 surface expression was determined in the entire PBMC fraction as well as on CD4^+^, CD8^+^, CD19^+^, and CD69^+^ cells from PBMCs of 72 healthy Ghanaian donors. Plots on the left show the median fluorescence intensity (MFI), plots on the right represent the mean percentage of CD95^+^ cells in each fraction of the donors stratified by c.−436C>A genotype. Horizontal lines indicate the median for each group.

Quantitative differences in CD95 surface expression in terms of c.−436C>A genotypes were assessed by staining PBMCs for CD95 from donors of the three genotypic groups. Each PBMC fraction was double-labeled in order to quantify CD95 expression on selected cell types, including CD4^+^, CD8^+^, CD19^+^, and CD69^+^ cells. Expression levels defined by the median fluorescent intensity (FI) for CD95^+^ cells were similar among the three genotypic groups for both, the entire PBMC fractions as well as the differentiated subpopulations (Figure 3). However, when examining the proportion of cells expressing CD95 on their surfaces, donors homozygous for the A allele showed a significantly higher percentage of CD69^+^CD95^+^ cells than those with heterozygous c.−436AC genotypes (Mann-Whitney test p = 0.027, Hodges-Lehmann 95% CI 2.5–45.2) or homozygous c.−436CC genotypes (p = 0.048, 95% CI 2.3–47.0) (Figure 3). No difference was found between the latter two (p = 0.978, 95% CI −15.1–19.6).

## Discussion

Collectively, our results show that the variant c.−436C>A in the promoter region of the *FAS* gene was associated with protection from severe malaria in Ghanaian children. No differences were found as to the major clinical forms of the disease. Statistical analyses resulted in an OR of 0.71 for severe malaria collectively, and ranged between 0.68 and 0.73 for distinct clinical phenotypes comprising severe malaria anemia, cerebral malaria, and other forms of severe malaria, which in our study included hyperlactatemia, prostration, and hyperparasitemia.

It is important to note, however, that the data presented solely allow the conclusion that c.−436C>A is a marker for a haplotype containing one or more genetic variants which reduce the risk of acquiring severe malaria. They do not show that c.−436C>A itself exerts this function. c.−436C>A is not predicted to directly affect a transcription factor binding site (TRANSFAC 7.0, http://www.gene-regulation.com/pub/databases.html). We did not obtain any evidence for additional variants which might be the causal ones by re-sequencing of the genomic region and searching for other variants which are linked to c.−436C>A and might have a stronger association with the phenotypes studied. However, re-sequencing was limited to 46 individuals and the linkage analyses were restricted to a sequence segment of 1 kb and to variants with an MAF of greater than 5%. Therefore, further association signals could have remained undiscovered and additional efforts are needed to convincingly identify the causal variant. c.334+46C>T, an intronic *FAS* variant which was associated with marginal significance, was not linked to −436C>A and followed a discordant model of inheritance.

A first hint as to the functional effect of the associated genetic variant came from a FACS analysis of PBMCs. Studying apparently healthy donors of the same ethnicity as the participants of the genetic study, the *FAS* product CD95 was found expressed on CD69^+^ cells of c.−436AA individuals in a substantially higher proportion of cells than in those from individuals carrying c.−436CA or c.−436CC. These data appear not to be in full agreement with the additive gene effect observed in the association study.

CD69 is a cell surface glycoprotein that is considered to be the earliest inducible molecule acquired during lymphoid activation. It is involved in lymphocyte proliferation and functions as a signal-transmitting receptor in lymphocytes, natural killer (NK) cells, and platelets [20]. With regard to malaria, *in vitro* experiments have shown that CD69 is universally up-regulated on NK cells in response to live intact *P. falciparum*-infected erythrocytes [21]. Thus, the FACS data support the notion that individuals homozygous for the A allele have a higher susceptibility to AICD through a CD95/CD95L interaction. In this experiment PBMCs were used which had been isolated from individuals who appeared healthy by the time their blood was taken. Therefore, it is conceivable to assume that heterozygous c.−436CA individuals have a small increase in the baseline CD95^+^ cell fraction that was not detectable in the experiment but, during malaria episodes, when CD95 expression is up-regulated [15], [16], would show an intermediate phenotype consistent with the additive effect found in the genetic association study.

Speculating about a possible mechanism underlying the observed association, it is worth mentioning that in our study the c.−436C>A variants were not associated with differences in parasite counts, neither among our patients during a severe malaria episode nor in a longitudinal observation of 390 Ghanaian children monitored by weekly examinations over 31 weeks. In this cohort, c.−436C>A was not associated with differences in the frequency of mild malaria episodes either, although the latter finding has to be interpreted with caution because of a moderate power to detect significant differences. Nevertheless, the mechanism underlying the c.−436C>A association presumably affects the pathogenesis of the severe forms of malaria. It appears most likely to assume that an increased expression of CD95 associated with c.−436AA and possibly c.−436CA facilitates the programmed cell death of lymphocytes upon immune activation. Thereby the protective FAS allele could alleviate immunopathology. As various studies have shown, immunopathology contributes substantially to the pathogenesis of severe malaria episodes [22], [23]. Several mechanisms have been proposed to contribute to the pathogenesis of malaria anemia including both, the destruction and decreased production of erythrocytes. Host mechanisms involved in the suppression of erythropoiesis may involve an excessive innate immune response with a persistent production of proinflammatory cytokines [24]. It is possible to envisage a role of CD95 in a down-regulation of cytokine-producing cells through apoptosis, which may alleviate an inhibition of erythropoiesis. Further studies are needed to support these hypotheses and to delineate how they apply to the various forms of malaria complications.

With allele frequencies of 0.12 in the present study population, 0.16 in the Yoruba from Nigeria, and 0.09 in African Americans, the A allele has exclusively been found in African populations or those with African ancestry and appears to be absent in Asian and European populations. It is possible to envisage a benefit for individuals carrying the A allele in regions endemic for malaria, which therefore might undergo a positive selection process maintaining the allele in a population. Supportive evidence for this evolutionary aspect comes from a genome-wide search for variants subjected to protozoa-driven selective pressure [25]. In that study, *FAS* was identified to be among the genes with at least one SNP associated with protozoan diversity in 52 human populations distributed worldwide.

Although the protective A allele may have an advantageous effect, with a MAF of 0.12 it appears to be maintained at a relatively low frequency in a population constantly exposed to *P. falciparum* infections. It is possible that its beneficial effect during severe malaria is counterbalanced by an adverse effect of the same allele in other infections. For instance, in patients with AIDS, a disease with too much apoptosis, the depletion of CD4^+^ T helper cells has been shown to be mediated by CD95 [26], [27]. In that case the presence of the A allele could lead to a higher apoptosis rate of these cells causing a more rapid loss of peripheral CD4^+^ T helper cells in these patients.

In summary, our study provides a rationale for a more detailed functional characterization of the *FAS* promoter region, in particular concerning the relevance of the polymorphism c.−436C>A in gene regulation with respect to *P. falciparum* infections. Further analysis of the CD95/CD95L signaling as part of the immune response to *P. falciparum* may yield further insights into the pathogenesis of life-threatening childhood malaria.

## Methods

### Ethics statement

The studies were approved by the Committee for Research, Publications and Ethics of the School of Medical Sciences, Kwame Nkrumah University of Science and Technology, Kumasi, Ghana.

Informed consent was obtained from parents or guardians of all participants for both case-control study groups, and from both parents of families after all procedures were explained in the local language.

### Severe malaria case-control sample sets

The present study was carried out in an area highly endemic for falciparum malaria in the Ashanti Region of Ghana, West Africa.

The first case-control sample set comprised 1195 severe malaria cases and 769 controls and had previously been subjected to genome-wide SNP genotyping utilizing the Affymetrix Genome-Wide Human SNP Array 6.0 (Affymetrix Inc., Santa Clara, USA) (manuscript in preparation). In the herein presented candidate-gene study data for SNPs included in the genome-wide chip were retrieved for the *FAS* gene locus. In addition, five selected variants based on sequencing results (see below) were genotyped in the initial study group.

The second, independent sample set derived from the same geographical area included 1412 cases and 2659 controls and was used for the replication of significant results for variant c.−436C>A (rs9658676) observed in the first study.

See Table 2 for a summarized characterization of the sample sets.

**Table 2:** Characteristics of severe malaria case-control sample sets.

	Median age (months, range)	Ethnic group (%)			
		Akan	Northerner	Ewe	n
**Case-control set 1**					
Severe Malaria	18 (3–108)	65.4	32.4	2.2	1195
Controls	21 (7–117)	69.6	28.3	2.1	769
Cases stratified by major phenotypes					
Severe Anemia^a^	18 (3–108)	64.3	33.6	2.1	897
Cerebral Malaria^b^	21 (5–108)	66.6	31.5	1.9	371
**Replication case-control set**					
Severe Malaria	20 (2–117)	69.8	26.8	3.4	1412
Controls	24 (7–912)	71.1	21.0	7.9	2659
Cases stratified by major phenotypes					
Severe Anemia^a^	15 (2–114)	73.9	22.7	3.4	729
Cerebral Malaria^b^	28 (7–109)	65.7	30.3	4.0	198
Others^c^	27 (5–117)	65.4	30.9	3.7	518

a Severe malaria anemia (hemoglobin level <5 g/dl) with or without additional complications including cerebral malaria;

b Cerebral malaria (BCS<3) with or without additional complications including severe anemia;

c Severe malaria complications not including severe anemia or cerebral malaria (hyperlactatemia, 100%; prostration, 83%; hyperparasitemia, 56%; acidosis, 54%; respiratory distress, 27%).

As previously described, all severe malaria patients were enrolled at the Komfo Anokye Teaching Hospital, Kumasi, between 2001 and 2005 in parallel with the “Severe Malaria in African Children” study [28]. Briefly, children aged between 6 and 120 months were included in the study if their Giemsa-blood smear was found to be positive for asexual *P. falciparum* parasites and either of the following clinical findings was diagnosed: (i) level of consciousness according to the Blantyre Coma Score (BCS) <3; (ii) hemoglobin concentration <5 g/dl; (iii) lactate concentration >5 mmol/L [29]. Parasite densities were recorded for 200 leukocytes and calculated assuming a leukocyte count of 8,000 per µl blood [30]. Controls in the initial case-control study comprised apparently healthy children who were from the same geographic area and were matched to the case group for age. In addition to apparently healthy children, the controls in the replication study included approximately 10% adults from the same area.

### Family-based study

In order to determine the impact of the variant c.−436C>A on *P. falciparum* density in mild childhood malaria, genotypes from an additional study with a total of 739 individuals comprising 390 siblings from 147 families were ascertained. DNA samples from parents and children used here were recruited and prepared as part of a genome-wide linkage analysis conducted in the Asante Akim North District, Ashanti region, Ghana [19]. The phenotype definitions for enrolled children were based on detailed weekly assessments over a period of 7 months during the raining season in 2002. Following WHO recommendations [31], mild malaria attacks were defined by either assessing fever (tympanic temperature of >37.7°C), or reported fever within the previous 4 days and a positive blood smear for asexual forms of *P. falciparum*. For each individual, the number of mild malaria episodes during the 31 week observation period was counted, whereby multiple episodes within 3 weeks were counted as one, as they were considered as recrudescences. Among the 390 siblings, a total of 504 malaria episodes were counted during the entire follow-up. Assuming a leukocyte count of 8,000 per µl blood parasite counts were recorded per 200 leukocytes (if >10 parasites/200 leukocytes) or 500 leukocytes (if ≤10 parasites/200 leukocytes). With a median of 32 parasites per µl blood, parasite densities ranged from 0–317,360/µl blood. Due the fact that the median point prevalence of malaria parasites was 54%, the 75^th^ percentile of parasite densities was considered a representative value of parasite density for each individual.

### Variant discovery in *FAS* regulatory and coding regions

Variants located in the coding regions of the *FAS* gene were detected by re-sequencing the 5′-UTR, the exons, including intron/exon boundaries, and the 3′-UTR of 46 genomic DNA samples drawn from study participants. In order to capture common alleles in the population, 23 DNA samples were selected from the control group. In addition, 23 genomic DNA samples of severe malaria cases were re-sequenced to cover information about possibly selected alleles which confer excess risk for severe malaria. With 23 samples, the probability of observing a variant with a MAF≥0.05 in a study group is 90% even if HWE does not hold [32].

Genomic DNA samples were amplified by PCR using primers that captured a 1000 bp region before the transcription start, the exonic sequences, including 30 bp of their intronic flanking regions, and 600 bp of the 3′-UTR. Oligonucleotides were designed using the Primer3 web-interface (http://frodo.wi.mit.edu/primer3/) against the reference sequence (NCBI NT_030059.13, Transcript NM_000043.3). Sequences of oligonucleotides and PCR conditions are listed in Table S3. After purifying the amplicons using Sephadex G-50 (Millipore GmbH, Schwalbach/Ts., Germany), each PCR fragment was subjected to a sequencing reaction according to the manufactures' instruction using the BigDye Terminator v3.1 Cycle Sequencing Kit (Applied Biosystems, Darmstadt, Germany). Electrophoresis was performed on the ABI PRISM 3100 DNA Analyzer. Assembly of generated sequences against the reference sequence and SNP detection was carried out using the SeqScape Software v2.5 (Applied Biosystems, Darmstadt, Germany). The presence of singletons was validated by re-sequencing PCR fragments from both ends.

### Genotyping *FAS* variants

Following the re-sequencing procedure detected variants were evaluated and selected for genotyping in the first case-control study (n = 1964). Selection criteria were an estimated MAF of ≥0.05 in at least one of the two groups, cases or controls, in addition to one of the following attributes. (i) The polymorphism leads to a non-synonymous amino-acid exchange in the receptor. (ii) The polymorphism is known to have functional relevance based on literature entries. (iii) The difference in estimated MAF from the re-sequencing between cases and controls is >10%. In agreement with these conditions a set of 5 SNPs, rs1800682, rs9658676, rs3218619, rs3218621, and rs3218614 were genotyped in the initial sample set of 1195 severe malaria cases and 769 controls.

In order to extract DNA for genotyping, blood samples were drawn from all participants. 0.5–1 ml blood was collected into citrate, and, before subjected to DNA extraction, the granulocyte fraction obtained from density gradient centrifugation was preserved in 4-M urea. DNA was extracted according to the suppliers' instructions (Nucleo-Mag 96 Blood; Macherey-Nagel, Düren, Germany). Prior to genotyping whole-genome amplification (WGA) using 10 ng DNA of each sample was conducted (GenomiPhi HY DNA Amplification Kit, GE Healthcare, Braunschweig, Germany). SNPs were analyzed by allele-specific hybridization in a melting curve analysis based on fluorescence resonance energy transfer (FRET) in a LightTyper device (Roche Diagnostics, Mannheim, Germany). Oligonucleotides and PCR conditions can be found in the Table S4.

### Genetic data analyses

For individuals part of the genome-wide association (GWA) study genotypes were retrieved for SNPs that are included in the Affymetrix SNP chip and are located at the *FAS* gene locus or surrounding regions of 10 kb adjacent to start and end of the gene. These were then merged with genotypes from the LightTyper platform. Genotypes were called using Birdseed, and standard quality control procedures comprised the exclusion of (i) individuals with SNP-call rates below 0.96, (ii) related individuals with an IBD>12.5% or (iii) individuals with heterozygosity rates <26% or >31%. The cluster plots of individual SNPs were verified by visual inspection. SNPs with call rates <96% were excluded. As part of the standard quality control for the five polymorphisms genotyped on the LightTyper platform, individuals with more than 50% missing genotypes were excluded, and SNPs were allowed to have a maximum of 4% missing genotypes.

Hence, a genotypic data set comprising a total of 19 SNPs at the *FAS* gene locus (Chr.10: 90,744,325–90,779,097; NCBI human genome Build 37.1) spanning approximately 35 kb was analyzed using primarily PLINK v1.07 [33]. For each polymorphism, the exact test was used to calculate HWE statistics [34]. The Cochran-Armitage trend test and logistic regression analyses were performed with additive coding of genotypes for the initial case-control study. Potential confounding factors, age, sex, and ethnicity were used as covariates in the logistic regression framework. In order to account for multiple testing, empirical significance values were ascertained by using the maxT permutation procedure with 10,000 permutations for each tested model, specifically for the additive, dominant, and recessive advantage. Empirical p-values (p_empirical_)<0.05 were considered significant. After a significant result was obtained, genotypes for variant c.−436C>A were retrieved in the replication set. We calculated results in the replication sample set by logistic regression accordingly and combined the results using the inverse variance weighted fixed effects model.

For each SNP a partitioning χ^2^-test for heterogeneity of ORs based on stratification into ethnic groups was carried out in order to detect possible population stratification.

The most plausible genetic model for variant c.−436C>A was selected using the approach of Bagos [35]. Based on a logistic regression model this approach uses a Wald test in order to test the null hypothesis of equality of the logORs associated with each genotype.

Linkage disequilibrium in the gene region was assessed using Haploview v4.1 for visualization of pairwise r^2^ values [36]. Association tests of haplotypes were calculated using the Haplostats package v1.4.4 in R [37] based on genotypes for all 19 SNPs in the GWA group of individuals (n = 1964). Score tests were carried out on full inferred haplotypes with frequencies >5% for the additive, dominant and recessive mode of inheritance, as well as on sub-haplotypes, using a sliding window of three SNPs. Age, sex, and ethnicity of individuals were used as covariates in the score statistics.

For variant c.−436C>A, the family-based qTDTs were done for the number of mild malaria attacks and the 75^th^ percentile of parasite density using the orthogonal model implemented in QTDT v2.5.1 [38]. The power calculations for the qTDT analysis are based on a MAF of 0.12 and a significance level of 0.05 [39]. It was estimated that c.−436C>A genotypes accounted for 5% of variance in the number of malaria episodes and 14% of the variance in the 75^th^ percentiles of parasite density.

### CD95 surface expression of peripheral blood mononuclear cells

PBMCs of 72 naïve adult African individuals from the Ashanti region in Ghana were investigated for their level of CD95 surface expression in relation to the c.−436C>A genotype. Prior to specific antibody-labeling and FACS of cells genotypes for c.−436C>A for all 72 individuals were gained as described above. In order to isolate the PBMC fraction from peripheral blood of donors a 2.5 ml of citrate blood diluted with 2.5 ml RPMI was subjected to Ficoll gradient centrifugation at 4°C. The lymphocyte layer was collected and washed twice with RPMI before PBMCs were stored in freezing medium, RPMI/10% fetal calf serum (FCS)/10%DMSO/1×Penicillin/Strepto-mycin at −80°C for 12 hours and subsequently in liquid nitrogen for long-term storage.

After thawing cell fractions were washed with RPMI medium and re-suspended in 2 ml of pre-chilled blocking solution (1×PBS/10% FCS/10% mouse serum). PBMCs were stained with biotinylated mAb against CD95 (anti-APO-1 IgG_1_ isotype, P.H. Krammer, Heidelberg, Germany) as well as with mAbs against CD4, CD19, CD8, and CD69 (BD Pharmingen, Heidelberg, Germany) for differentiation of lymphocyte subsets. Biotin-conjugated mouse IgG_1_ antibody (BD Pharmingen) was used as an isotype control. FACS Canto II flow cytometer and FACS-Diva software (BD Biosciences, San Jose, USA) were used to perform analyzes of the cells. As cell counts varied significantly with the donors, a minimum of 10,000 analyzed cells per assay was set for a sample to be included in the analysis.

The surface expression level of CD95 was assessed in two different ways, first by directly measuring FI of cells expressing CD95 and second by defining the percentage of CD95^+^ cells in particular cell fractions. The average percentage of CD95^+^ cells and the median FI of donors were compared among three genotypic groups of donors (c.−436CC, n = 24; AC, n = 22; and AA, n = 26) using the Mann-Whitney test at the nominal 5% test-level together with corresponding Hodges-Lehmann 95% confidence intervals.

## Supporting Information

Figure S1 Score statistic global p-values for additive effect of sub-haplotypes at the *FAS* gene locus. Haplotypes tested for association in a sliding window comprising three SNPs.
(TIFF)

Table S1 Variants in regulatory and coding regions of *FAS* from genomic DNA of 23 controls and 23 severe malaria cases identified by re-sequencing. ^a^Position in transcript NM_000043.3. ^b^Alleles are matched to the forward strand.
(DOC)

Table S2 Haplotype-specific p-values from score association tests with severe falciparum malaria based on inferred haplotypes with frequencies >5% at the *FAS* locus. ^a^p-values adjusted for gender, age, and ethnicity.
(DOC)

Table S3 Oligonucleotides and PCR conditions for re-sequencing of regulatory and coding regions of *FAS* from genomic DNA. Each reaction mixture contained 10 ng of genomic DNA, 1×PCR Buffer BD, 1 U FIREPol DNA polymerase I (Solis BioDyne, Estonia), 200 µM of each dNTP, 1 µM of each PCR Primer, above stated concentration of MgCl_2_, and water to a final volume of 20 µl. PCR conditions were as follows: 94°C for 3 min, 40 cycles of 95°C for 1 min, specific annealing temperature for 1 min, 72°C for 1 min followed 72°C for 10 min.
(DOC)

Table S4 Oligonucleotides and PCR conditions for genotyping selected *FAS* variants. Each reaction mixture contained 2 µl of a 1∶200 dilution of whole-genome wide amplified genomic DNA, 1×PCR Buffer BD, 1× Solution S, 1 U FIREPol DNA polymerase I (Solis BioDyne. Estonia), 200 µM of each dNTP, 1 µM or 0.2 µM of each PCR primer, 0.2 µM of each fluorescence-labeled primer, above stated MgCl_2_. Concentration, and water to a final volume of 10 µl. PCR conditions were as follows: 95°C for 3 min. 45 cycles of 95°C for 1 min. 55°C for 1 min. 72°C for 1 min, followed by 72°C for 10 min.
(DOC)
