# Supplementary figures and images for: A −436C>A Polymorphism in the Human FAS Gene Promoter Associated with Severe Childhood Malaria
Source: PLoS Genet. 2011 May 19;7(5):e1002066. doi: 10.1371/journal.pgen.1002066 (PMC3098189; doi:10.1371/journal.pgen.1002066)

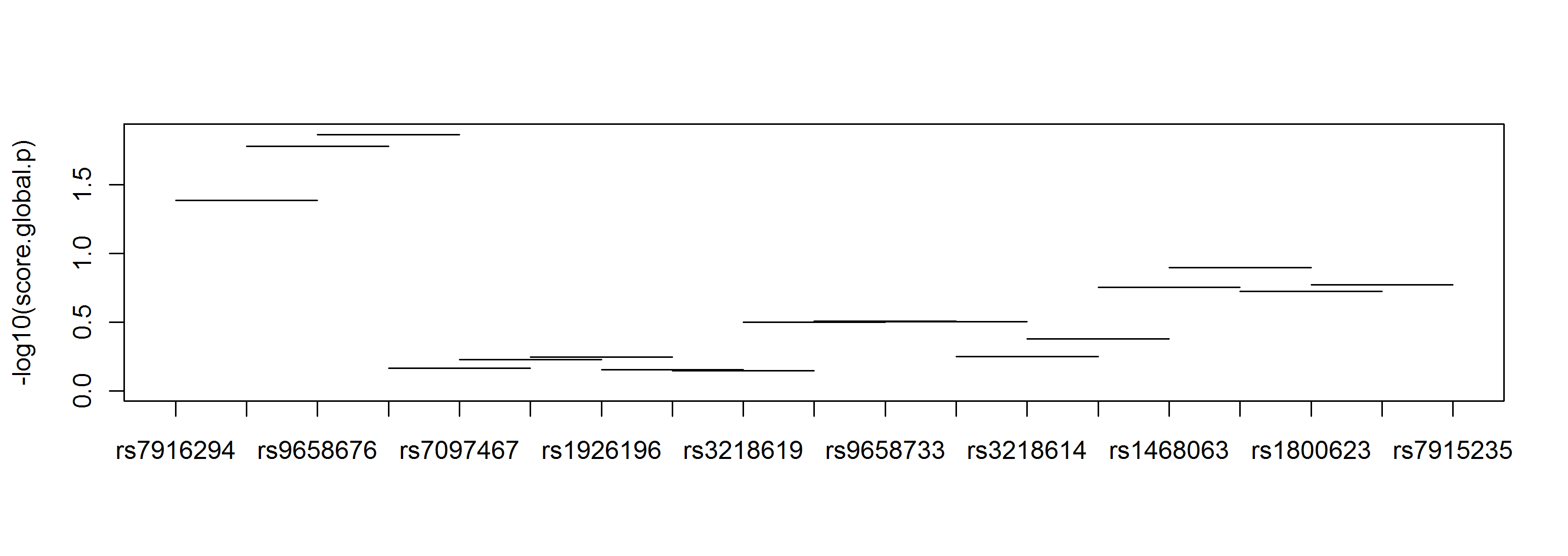

Supplement: Figure S1 — Score statistic global p-values for additive effect of sub-haplotypes at the FAS gene locus. Haplotypes tested for association in a sliding window comprising three SNPs. (TIFF) [file pgen.1002066.s001.tif]
