# Supplementary material for: A −436C>A Polymorphism in the Human FAS Gene Promoter Associated with Severe Childhood Malaria
Source: PLoS Genet. 2011 May 19;7(5):e1002066. doi: 10.1371/journal.pgen.1002066 (PMC3098189; doi:10.1371/journal.pgen.1002066)
Supplement: Table S1 — Variants in regulatory and coding regions of FAS from genomic DNA of 23 controls and 23 severe malaria cases identified by re-sequencing. aPosition in transcript NM_000043.3. bAlleles are matched to the forward strand. (DOC) [file pgen.1002066.s002.doc]

Table S1. Variants in regulatory and coding regions of *FAS* from genomic DNA of 23 controls and 23 severe malaria cases identified by re-sequencing.

| SNP ID | Position^a^ | Alleles^b^ | Amino acid exchange | Estimated MAF Severe Malaria Cases | Estimated MAF Controls |
| --- | --- | --- | --- | --- | --- |
| rs1800682 | c.-671 | A/G |  | 0.21 | 0.21 |
| rs9658676 | c.-436 | C/A |  | 0.04 | 0.2 |
| rs12251390 | c.-99 | G/A |  | 0.02 | 0.02 |
| rs2274355 | c.-95 | G/A |  | 0.19 | 0.21 |
| rs9658678 | c.-56 | C/- |  | 0.04 | 0.04 |
| rs5030766 | c.-34 | A/G |  | 0.15 | 0.11 |
| rs3218619 | c.46 | G/A | A16T | 0.08 | 0.15 |
| rs3218621 | c.141 | G/A |  | 0.06 | 0.21 |
| rs2031611 | c.197-62 | G/C |  | 0.27 | 0.13 |
| rs3218614 | c.365 | C/T | T122I | 0.02 | 0.06 |
| rs2296600 | c.506-71 | C/G |  | 0.31 | 0.27 |
| rs28362322 | c.550 | A/G |  | 0.02 | 0 |
| rs2234978 | c.642 | C/T |  | 0.31 | 0.23 |
| rs9658774 | c.*142 | A/G |  | 0.10 | 0.19 |
|  | c.*978 | C/T |  | 0.02 | 0.02 |
| rs1468063 | c.*1084 | C/T |  | 0.27 | 0.27 |

^a^ Position in transcript NM_000043.3, ^b^ Alleles are matched to the forward strand
