# Supplementary material for: A −436C>A Polymorphism in the Human FAS Gene Promoter Associated with Severe Childhood Malaria
Source: PLoS Genet. 2011 May 19;7(5):e1002066. doi: 10.1371/journal.pgen.1002066 (PMC3098189; doi:10.1371/journal.pgen.1002066)
Supplement: Table S2 — Haplotype-specific p-values from score association tests with severe falciparum malaria based on inferred haplotypes with frequencies >5% at the FAS locus. ap-values adjusted for gender, age, and ethnicity. (DOC) [file pgen.1002066.s003.doc]

Table S2. Haplotype-specific p-values from score association tests with severe falciparum malaria based on inferred haplotypes with frequencies >5% at the *FAS* locus.

| Haplotype | rs7916294 | rs1800682 | rs9658676 | rs10509561 | rs7097467 | rs7097572 | rs1926196 | rs9658702 | rs3218619 | rs3218621 | rs9658733 | rs2296601 | rs3218614 | rs2234978 | rs1468063 | rs2862833 | rs1800623 | rs4934435 | rs7915235 | Frequency Cases N=1195 | Frequency Controls N=769 | p-value^a^ additive | p-value^a^ dominant | p-value^a^ recessive |
| --- | --- | --- | --- | --- | --- | --- | --- | --- | --- | --- | --- | --- | --- | --- | --- | --- | --- | --- | --- | --- | --- | --- | --- | --- |
| 1 | A | A | A | A | T | C | C | G | A | G | A | C | C | T | C | A | A | A | G | 0.05 | 0.06 | 0.076 | 0.066 | NA |
| 2 | A | G | C | T | T | C | C | G | G | G | A | C | C | T | C | A | A | A | G | 0.19 | 0.21 | 0.158 | 0.282 | 0.181 |
| 3 | A | G | C | A | T | C | C | A | G | G | A | C | C | C | C | G | G | G | G | 0.13 | 0.13 | 0.672 | 0.747 | 0.701 |
| 4 | C | A | C | A | T | T | T | G | G | G | A | C | C | C | C | G | G | G | G | 0.15 | 0.16 | 0.720 | 0.987 | 0.171 |
| 5 | C | G | C | A | C | C | C | G | G | G | T | T | C | C | T | A | G | G | A | 0.08 | 0.08 | 0.908 | 0.800 | 0.531 |

^a^ p-values adjusted for gender, age, and ethnicity
