# Supplementary material for: A −436C>A Polymorphism in the Human FAS Gene Promoter Associated with Severe Childhood Malaria
Source: PLoS Genet. 2011 May 19;7(5):e1002066. doi: 10.1371/journal.pgen.1002066 (PMC3098189; doi:10.1371/journal.pgen.1002066)
Supplement: Table S3 — Oligonucleotides and PCR conditions for re-sequencing of regulatory and coding regions of FAS from genomic DNA. Each reaction mixture contained 10 ng of genomic DNA, 1×PCR Buffer BD, 1 U FIREPol DNA polymerase I (Solis BioDyne, Estonia), 200 µM of each dNTP, 1 µM of each PCR Primer, above stated concentration of MgCl2, and water to a final volume of 20 µl. PCR conditions were as follows: 94°C for 3 min, 40 cycles of 95°C for 1 min, specific annealing temperature for 1 min, 72°C for 1 min followed 72°C for 10 min. (DOC) [file pgen.1002066.s004.doc]

Table S3. Oligonucleotides and PCR conditions for re-sequencing of regulatory and coding regions of *FAS* from genomic DNA.

| PCR | Oligonucleotides Forward/Reverse | Annealing Temperature [°C] | Concentration MgCl_2_ [mM] |
| --- | --- | --- | --- |
| FAS1 | GAAAATTGGCCAGGAAATAATG/GGGACTAAGACGGGGTAAGC | 55.0 | 3.0 |
| FAS2 | GACCTGCTGCTTTCTTGGAG/GGGGAACCAAAAACTGTAAAA | 55.0 | 3.0 |
| FAS3 | CTCCCCTTGTGTTTTAGAAGAG/CCACAGTAGGCCCCAATTTC | 60.0 | 2.0 |
| FAS4 | TGGATCTCAAAAATCCATGC/GGCAAAGCAGGACTAGAACC | 60.0 | 3.0 |
| FAS5 | GAATACGTTTGCCAGAGATGC/ACCAAAGTGGTAGCTTTTTATAGG | 55.0 | 2.0 |
| FAS6 | GGTCTCCTGCGATGTTTGG/CAAGACTCCATCTCAAACAAAATG | 55.0 | 3.0 |
| FAS7 | CCTTCTTAATCACTTAATCTAGCTTCC/TGCTGAGCAGGTAGAATTGTATG | 60.0 | 2.0 |
| FAS8 | CTGGGTATATGGCAGGATTTG/AAGGTCTTTGAGGTAGAGCCT | 55.0 | 2.0 |
| FAS9 | TCTAAACTTTGTTTATAACTCTGAGAAG/GGGATCCAAGAAGCATTAAAGA | 60.0 | 2.0 |

Each reaction mixture contained 10 ng of genomic DNA, 1xPCR Buffer BD, 1 U FIREPol DNA polymerase I (Solis BioDyne, Estonia), 200 µM of each dNTP, 1 µM of each PCR Primer, above stated concentration of MgCl_2_, and water to a final volume of 20 µl. PCR conditions were as follows: 94°C for 3 min, 40 cycles of 95°C for 1 min, specific annealing temperature for 1 min, 72°C for 1 min followed 72°C for 10 min.
