# Supplementary material for: A −436C>A Polymorphism in the Human FAS Gene Promoter Associated with Severe Childhood Malaria
Source: PLoS Genet. 2011 May 19;7(5):e1002066. doi: 10.1371/journal.pgen.1002066 (PMC3098189; doi:10.1371/journal.pgen.1002066)
Supplement: Table S4 — Oligonucleotides and PCR conditions for genotyping selected FAS variants. Each reaction mixture contained 2 µl of a 1∶200 dilution of whole-genome wide amplified genomic DNA, 1×PCR Buffer BD, 1× Solution S, 1 U FIREPol DNA polymerase I (Solis BioDyne. Estonia), 200 µM of each dNTP, 1 µM or 0.2 µM of each PCR primer, 0.2 µM of each fluorescence-labeled primer, above stated MgCl2. Concentration, and water to a final volume of 10 µl. PCR conditions were as follows: 95°C for 3 min. 45 cycles of 95°C for 1 min. 55°C for 1 min. 72°C for 1 min, followed by 72°C for 10 min. (DOC) [file pgen.1002066.s005.doc]

Table S4. Oligonucleotides and PCR conditions for genotyping selected *FAS* variants.

| Variant | | Oligonucleotides | | | Sequence | Primer ratio (forward:reverse) | | Concentration MgCl2 [mM] | |
| --- | --- | --- | --- | --- | --- | --- | --- | --- | --- |
|  | rs1800682 | | FAS_976_rev | TCAGAGAAAGACTTGCGGG | | | 1:5 | | 2.0 |
|  |  | | FAS_976_for | TGCGATTTGGCTTAAGTTGTTAG | | |  | |  |
|  |  | | FAS_976_sen | GTTAACTGTCCATTCCAGGAAC-FLU | | |  | |  |
|  |  | | FAS_976_anc | CY5-CTGTGAGCCTCTCATGTTGCAG-PHO | | |  | |  |
|  | rs9658676 | | FAS_1211_for | CTCCTTCAAGACCTCCC | | | 1:5 | | 1.0 |
|  |  | | FAS_1211_rev | GGTTCGTTGCACAAATGG | | |  | |  |
|  |  | | FAS_1211_sen | TCCTCACCTGAAGTGAGCATGC-FLU | | |  | |  |
|  |  | | FAS_1211_anc | CY5-GCCACTGCAGGAACGCCCCG-PHO | | |  | |  |
|  | rs3218619 | | FAS_rs*8619_for | TGGGTTACACTTGTTTACCAC | | | 5:1 | | 3.0 |
|  |  | | FAS_rs*8619_rev | CTGTAGTAACAGTCTTCCTCAAT | | |  | |  |
|  |  | | FAS_rs*8619_sen | CY5-CTAGCAACAGACGTAAGAACCTGTAAAA-PHO | | |  | |  |
|  |  | | FAS_rs*8619_anc | GTCAGTCACTTGGGCATTAACACTTTTGGACGATA-FLU | | |  | |  |
|  | rs3218621 | | FAS6.2_sen | TCCAAGTTCTGAGTCTCAAC-FLU | | | 5:1 | | 2.0 |
|  |  | | FAS6.2_anc | CY5-GTAGTAACAGTCTTCCTCAATTCCAATCCC-PHO | | |  | |  |
|  |  | | FAS6.2_for | TGCCCAAGTGACTGACAT | | |  | |  |
|  |  | | FAS6.2_rev | CTGGAGGACAGGGCTTAT | | |  | |  |
|  | rs3218614 | | FAS4_2_for | CATAGTCTGCTTATAATTAGCCG | | | 1:5 | | 2.0 |
|  |  | | FAS4_2_rev | CTTACTTGGTGCAAGGGTCA | | |  | |  |
|  |  | | FAS_rs*8614_sen | CY5-GGACCCAGAATACCAAGTGCA-PHO | | |  | |  |
|  |  | | FAS_rs*8614_anc | GATTTTCTAGGCTTAGAAGTGGAAATAAACTGCAC-FLU | | |  | |  |

Each reaction mixture contained 2 µl of a 1:200 dilution of whole-genome wide amplified genomic DNA, 1xPCR Buffer BD, 1x Solution S, 1 U FIREPol DNA polymerase I (Solis BioDyne. Estonia), 200 µM of each dNTP, 1 µM or 0.2 µM of each PCR primer, 0.2 µM of each fluorescence-labeled primer, above stated MgCl_2_. Concentration, and water to a final volume of 10 µl. PCR conditions were as follows: 95°C for 3 min. 45 cycles of 95°C for 1 min. 55°C for 1 min. 72°C for 1 min, followed by 72°C for 10 min.
